# ULaMDyn: enhancing excited-state dynamics analysis through streamlined unsupervised learning

**DOI:** 10.1039/d4dd00374h

**Published:** 2025-01-08

**Authors:** Max Pinheiro, Matheus de Oliveira Bispo, Rafael S. Mattos, Mariana Telles do Casal, Bidhan Chandra Garain, Josene M. Toldo, Saikat Mukherjee, Mario Barbatti

**Affiliations:** a Aix Marseille University, CNRS, ICR 13397 Marseille France maxjr82@gmail.com bidhan-chandra.garain@univ-amu.fr; b Department of Chemistry, Physical Chemistry and Quantum Chemistry Division, KU Leuven 3001 Leuven Belgium; c UCBL, ENS de Lyon, CNRS, LCH UMR 5182 69342 Lyon Cedex 07 France; d Faculty of Chemistry, Nicolaus Copernicus University in Toruń Gagarina 7 87-100 Toruń Poland; e Institut Universitaire de France 75231 Paris France mario.barbatti@univ-amu.fr https://barbatti.org/

## Abstract

The analysis of nonadiabatic molecular dynamics (NAMD) data presents significant challenges due to its high dimensionality and complexity. To address these issues, we introduce ULaMDyn, a Python-based, open-source package designed to automate the unsupervised analysis of large datasets generated by NAMD simulations. ULaMDyn integrates seamlessly with the Newton-X platform and employs advanced dimensionality reduction and clustering techniques to uncover hidden patterns in molecular trajectories, enabling a more intuitive understanding of excited-state processes. Using the photochemical dynamics of fulvene as a test case, we demonstrate how ULaMDyn efficiently identifies critical molecular geometries and critical nonadiabatic transitions. The package offers a streamlined, scalable solution for interpreting large NAMD datasets. It is poised to facilitate advances in the study of excited-state dynamics across a wide range of molecular systems.

## Abstract 2

ULaMDyn automates the unsupervised analysis of high-dimensional nonadiabatic molecular dynamics data, enabling the discovery of key properties critical for excited-state dynamics.

## Graphical abstract

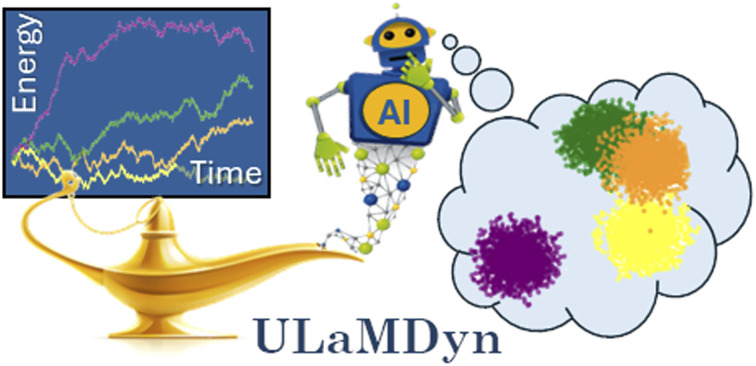

## 1 Introduction

Photochemical and photophysical phenomena in molecules, supramolecular assemblies, and solids involve the time evolution of electronic populations through multiple electronic states. Understanding these processes requires nonadiabatic dynamics simulations that account for the interplay between nuclear and electronic motions beyond the adiabatic approximation.^1–6^ Given the high computational costs of these simulations, several strategies have been developed. One approach is to address the problem fully quantum mechanically but with reduced dimensionality, such as focusing only on electron dynamics within a fixed nuclear framework or considering a few nuclear modes. Another strategy is to retain full dimensionality by treating part of the system's degrees of freedom quantum mechanically and the rest classically. This latter approach underpins Nonadiabatic Mixed Quantum-Classical (NAMQC) dynamics. NAMQC dynamics is a broad category that includes various methods developed over the years to account for time-resolved simulations.^7–16^ Among these, trajectory surface hopping (TSH) is the most widely used.^17^ In this approach, a swarm of independent trajectories is propagated, each utilizing the forces from a single adiabatic electronic state. The nonadiabatic nature of the dynamics is captured by allowing the trajectories to probabilistically hop to different electronic state surfaces.

Trajectory-based nonadiabatic dynamics require running numerous independent trajectories to approximate the quantum system behavior until statistical convergence is achieved. At each time step of the dynamics, electronic properties for a given molecular configuration are computed. Thus, statistical analysis provides essential insights into dynamics features, such as excited-state lifetimes, reaction channel branching ratios, and dominant molecular motions. With the advancements in surface hopping techniques and the substantial growth of computational power, the systems under study are becoming increasingly complex.^18,19^ This progress allows for the generation of a large number of configurations more efficiently, resulting in a massive amount of high-dimensional data and reducing the uncertainty in the calculated mean properties.

When considering nonadiabatic molecular dynamics (NAMD) for data generation, a sequential array of frames representing diverse molecular configurations is produced. These frames can be condensed into vectors, enriched with quantum properties computed during the dynamics, creating a comprehensive dataset of molecular behaviors. As time scales extend, the dataset becomes increasingly extensive and intricate. Moreover, multiple electronic states and their associated potential energy surfaces are involved in NAMD dynamics. As molecular trajectories evolve over time, they encounter regions of stronger nonadiabatic coupling, where transitions between states can occur. The surface hopping approximation adds complexity by increasing data volume through the requirement of multiple independent trajectories, which in turn further increases system dimensionality. Given the complexity of these dynamics and the high-dimensional nature of molecular geometry data, advanced statistical methods like dimensionality reduction and unsupervised learning are necessary to extract meaningful patterns. ULaMDyn addresses this challenge by automating the analysis of these large datasets, uncovering critical internal coordinates, and allowing researchers to identify key features across molecular trajectories. This concept has already been successfully applied to the analysis of ground-state molecular dynamics data, demonstrating its potential for broader applications.^20–28^

However, the application of unsupervised learning algorithms to analyze nonadiabatic dynamics simulation results remains challenging, with only limited progress made in recent years. Perrella *et al.* applied K-medoids clustering techniques to molecular dynamics (MD) trajectories to reduce the dataset size for *ab initio* modeling of electronic absorption spectra.^29^ They tested this approach on two challenging case studies: a non-covalent charge-transfer dimer and a ruthenium complex in solution at room temperature. Virshup *et al.* utilized the diffusion map technique for dimensionality reduction in analyzing photoisomerization dynamics through *ab initio* multiple spawning simulations.^30^ Similarly, Belyaev *et al.* applied the same dimensionality reduction method to examine the geometric evolution in TSH nonadiabatic dynamics.^31^ Li *et al.* explored the geometric evolution in nonadiabatic dynamics using two closely related dimensionality reduction techniques: classical multidimensional scaling (MDS) and isometric feature mapping (ISOMAP), alongside the density-based spatial clustering of applications with noise (DBSCAN) clustering approach.^32,33^ Principal component analysis (PCA) was employed by Peng *et al.*^34^ and Capano *et al.*^35^ to study the photophysics of a Cu-complex in TSH dynamics and to investigate the role of bath motion in the symmetrical quasi-classical dynamics method based on the Meyer–Miller mapping Hamiltonian, respectively.

Recently, Lan *et al.* have developed a hierarchical protocol using unsupervised machine learning to automatically identify different photoreaction channels and critical molecular motions from on-the-fly TSH dynamics simulations, effectively addressing challenges like characterizing the ring distortion for keto isocytosine.^36,37^ Lan and co-worker's recent work highlights the growing potential of integrating unsupervised machine learning methods into NAMD simulation analysis, emphasizing the need for interdisciplinary collaboration and the future development of automated “black-box” tools to enhance efficiency and insight into nonadiabatic dynamics.^38^ Acheson *et al.* have introduced clustering as a computational tool for interpreting photoexcited dynamics trajectories, using variance mapping, L_2_-norm, and dynamic time warping (DTW) measures with the DBSCAN algorithm to classify complex trajectory datasets in an unbiased manner, showcasing its application in a photochemical ring-opening reaction.^39^ Additionally, some progress has been made in applying unsupervised machine learning algorithms to analyze the nonadiabatic dynamics of solid-state systems.^40^ Prezhdo *et al.* applied unsupervised machine learning to analyze correlations between structural and electronic properties of CsPbI_3_ perovskite, establishing key geometric features and motions that govern charge carrier dynamics in this widely studied solar cell material.^41^ Despite the emergence of such advanced techniques to address the challenges of analyzing high-dimensional data in this fast-paced field, these methods often function independently, requiring distinct approaches for each type of data generation and clustering. This separation can complicate the practical analysis and interpretation of results.

**Fig. 1:**
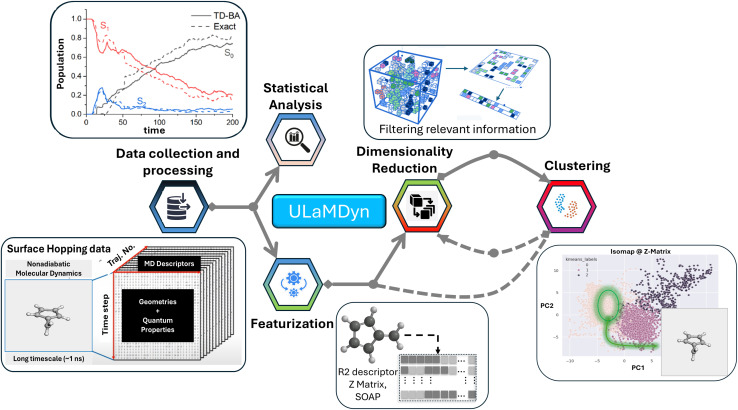
ULaMDyn streamlines the analysis of high-dimensional data from Nonadiabatic Molecular Dynamics (NAMD) simulations. It integrates statistical analysis, dimensionality reduction, and clustering to extract relevant information and visualize key molecular properties, simplifying the understanding of complex trajectories.

To address this challenge, we developed a unified, free, and open-source Python package called ULaMDyn, which stands for “Unsupervised Learning Analysis of Molecular Dynamics” (Fig. 1). It is designed to automate the discovery of hidden patterns in high-dimensional molecular datasets. ULaMDyn offers a comprehensive set of tools for preprocessing, statistical analysis, and unsupervised learning of trajectory data generated by Newton-X.^42^ Seamlessly integrated with Newton-X's surface hopping NAMD, it streamlines the processing and analysis of simulation outputs. The package also leverages dimensionality reduction and clustering techniques to enhance dataset construction for supervised learning tasks conducted using MLatom, which is similarly interfaced with Newton-X.^43^ This unified approach establishes a comprehensive pipeline that combines both supervised and unsupervised learning methodologies, thereby streamlining the analysis of molecular dynamics simulations and improving the interpretability and understanding of complex potential energy surfaces and nonadiabatic dynamics.

## 2 Prerequisites

To effectively engage with this article, it is recommended that the reader has a basic knowledge of Python. Familiarity with the nonadiabatic dynamics package Newton-X^42^ and an understanding of NAMD principles is beneficial, as our discussions incorporate NAMD data.^1^ Additionally, a basic understanding of fundamental machine learning concepts, such as evaluation metrics and data partitioning strategies, is assumed to grasp the more advanced topics discussed in this paper on unsupervised learning.

To run ULaMDyn, essential Python packages like SciPy, Pandas, and tslearn should be installed. The list of requirements is provided when downloading the package, and instructions for any additional libraries are provided in the Python notebook accompanying the package. The reader must have access to a platform capable of executing them, such as a dedicated integrated development environment (IDE) like Jupyter or equivalent alternatives. Alternatively, ULaMDyn can be run as a single code line, and subsequent analysis can be done using simple Python scripts. The notebook example and code provided in this article are designed to be executable on a standard laptop, ensuring accessibility and ease of implementation for a broad range of readers.

## 3 Methods

In the context of nonadiabatic dynamics, unsupervised learning techniques are often applied to various data types, including molecular geometries (3D structures), NAMD trajectories (time-series data), electronic properties (tabular data), geometric parameters of photochemical reaction intermediates (tabular data), and excited state interactions (graph networks). These techniques can generally be categorized into two groups:

(1) Dimensionality reduction schemes. They reduce the complexity of high-dimensional datasets by mapping them to lower-dimensional spaces, ensuring that essential information and features present in the data are preserved.

(2) Clustering techniques. They aim to identify groups of data points with shared attributes by detecting distinct patterns in the data, such as metastable states from an MD trajectory.

All these methods can make the exploration of large phase spaces more manageable and are essential tools for identifying photochemical pathways and relevant molecular geometries.

### 3.1 Molecular representations

An appropriate molecular representation is essential for capturing the relevant chemical variability in data, significantly influencing the accuracy and interpretability of machine learning models. This process includes descriptor and feature selection, where specific molecular attributes are identified and combined into a compact vector representation for each molecule.^44,45^

In ULaMDyn, there are three types of geometry-based descriptors, which incorporate translation and rotation invariances: the pairwise distances between atoms (R2 family of descriptors), the Z-matrix representation, and Smooth Overlap of Atomic Positions (SOAP) descriptors. Additionally, Cremer–Pople parameters for ring puckering analysis are also available, providing a detailed description of ring conformations.^46^

#### 3.1.1 R2 descriptor

The R2 descriptor is represented as a flattened matrix containing all pairwise Euclidean distances between atoms in a molecule. Since this matrix is symmetric with respect to the interchange of atom indices (*i.e.*, *D*_*ij*_ = *D*_*ji*_), only the lower triangular portion of the R2 matrix is included in the final dataset.

ULaMDyn also provides additional feature engineering steps to convert the R2 distance matrix into other meaningful variants:

• Inverse R2: this descriptor is defined as the inverse of the R2 (1/*R*_*ij*_) distance matrix, similar to the Coulomb matrix descriptor.^47^

• Delta R2: this descriptor represents the difference between the R2 vector of the current geometry at time *t* and the corresponding R2 vector of a reference geometry, typically the ground-state geometry (*R*_*ij*_(*t*) − *R*_*ij*_(ref)).

• RE: the RE descriptor is the R2 vector normalized relative to the reference geometry (*R*_*ij*_(ref)/*R*_*ij*_(*t*)).

#### 3.1.2 Z-Matrix

The Z-matrix is a structured way to represent the geometry of a molecule using internal coordinates rather than Cartesian coordinates. In a Z-matrix, the position of each atom is described by a combination of bond lengths, bond angles, and dihedral angles relative to other atoms in the molecule. This format inherently captures the connectivity and the relative spatial arrangement of atoms, and it naturally accommodates rotational and translational invariance. Thus, it is an efficient descriptor for capturing the essential structural features of molecules. Additionally, it simplifies the specification of molecular structures, especially when dealing with large systems or those with symmetrical properties. ULaMDyn provides quantities related to distances in angstroms, while features derived from angles are provided in degrees. Since the Z-matrix module in ULaMDyn provides separate functions to compute bond distances, angles, dihedrals, or even bending angles (six atoms) to describe large out-of-plane motions, the user has the possibility of augmenting the standard Z-matrix dataset by including other key variables relevant to the specific system/dynamics.

In addition to the standard Z-matrix, ULaMDyn also offers delta Z-matrix. The delta Z-matrix represents the difference between the Z-matrix of the current geometry at time *t* and the Z-matrix of a reference geometry. The delta Z-matrix is particularly effective for analyzing variations with respect to a reference geometry, such as the equilibrium geometry. Encoding deviations from a fixed structure facilitates the detection of structural distortions and the characterization of dynamic behaviors. We recommend using it for cases where deviations from a known equilibrium structure are of primary interest.

In the case of delta Z-matrix, additional non-linear transformations can be applied to the descriptor with the goal of achieving a better scaling between features while still emphasizing relevant structural variations. The available transformations are:

• tan h Z-matrix: it applies a hyperbolic tangent transformation to all features of the delta Z-matrix. This non-linear transformation is particularly useful to handle angular features, including bond angles and dihedral angles. Applying the hyperbolic tangent function ensures that angles and dihedrals are scaled smoothly and boundedly. It is well-suited for machine learning applications where unbounded values could cause numerical instability. We recommend using it when angular features play a significant role, particularly in systems with flexible torsional degrees of freedom.

• Sig Z-matrix: this non-linear transformation uses the sigmoid function to map distances, angles, and dihedral angles to a bounded range. This representation emphasizes smaller structural variations while down-weighting large deviations, as the sigmoid function compresses extreme values. Use it for applications requiring bounded feature representations, particularly when relative changes are more critical than absolute changes.

#### 3.1.3 Smooth overlap of atomic positions (SOAP)

Smooth Overlap of Atomic Positions (SOAP), as implemented in DScribe^48^ and utilized by ULaMDyn, is a descriptor that captures local atomic geometries by expanding a Gaussian-smeared atomic density using orthonormal functions derived from spherical harmonics and radial basis functions.^49^ In this implementation, SOAP requires the atomic coordinates in the form of *XYZ* data and the corresponding labels for each atom. Additionally, the SOAP constructor accepts various other parameters that allow for further customization, with detailed explanations available in the DScribe library documentation.^48^

The SOAP output is represented as the partial power spectrum vector ***p***, with its elements defined as follows^45^1
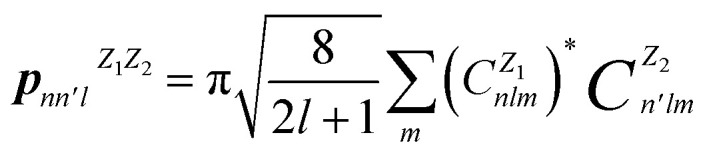
where *n* and *n*′ are indices for the different radial basis functions up to *n*_max_, *l* is the angular degree of the spherical harmonics up to *l*_max_·*Z*_1_ and *Z*_2_ are atomic species.

The coefficients 
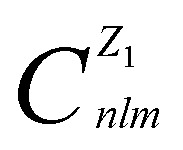
 are defined as the following inner products2
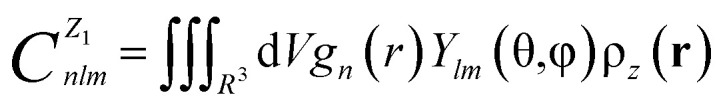
where *ρ*_*Z*_(**r**) is the Gaussian smoothed atomic density for atoms with atomic number *Z* defined as,3
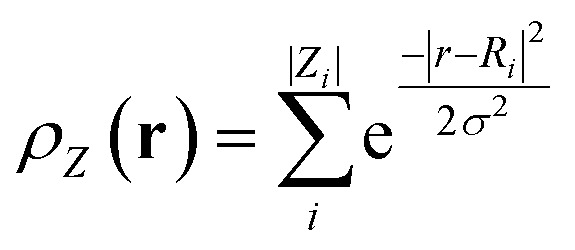
In eqn (2), *Y*_*lm*_(*θ*,*ϕ*) are the real spherical harmonics, and *g*_*n*_(*r*) is the radial basis function.

#### 3.1.4 Cremer–Pople parameters

Cremer–Pople puckering parameters provide a mathematical framework to describe the three-dimensional conformations of non-planar ring systems, which are particularly useful for cyclic organic molecules. For an *N*-membered ring (where *N* > 3), there are *N* − 3 ring-puckering coordinates that quantify deviations from planarity. For a six-membered ring, these coordinates reduce to three key parameters: the puckering amplitude *Q*, which measures the extent of puckering, and two angular variables, *θ* and *ϕ*, which describe the degree and type of distortion (*e.g.*, chair, boat, or twist). This formalism can be generalized to rings of different sizes, making it a versatile tool for analyzing ring conformations.

In the future, we plan to implement Faber–Christensen–Huang–Lilienfeld (FCHL),^50^ and Many-Body Tensor Representation (MBTR)^51^ descriptors in ULaMDyn.

### 3.2 Data preprocessing

Data preprocessing is a crucial step in any machine learning pipeline and requires as much attention as model development.^52^ Proper preprocessing converts raw data into a format suitable for model training, with a key aspect being the management of features with varying scales. Techniques like normalization and scaling are essential to address these issues. Normalization and scaling adjust the range and distribution of data features to enhance algorithm performance. Standardization, or *z*-score normalization, is a standard method that transforms data to have a mean of zero and a standard deviation of one, ensuring that features with different units or scales contribute equally to the model. The formula for *z*-score normalization is:4
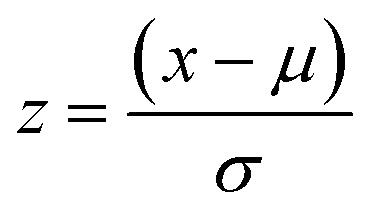
where *x* is the original value, *μ* is the mean of the feature, and *σ* is the standard deviation of the feature.

Distance-based methods, whether for dimensionality reduction or clustering, are susceptible to feature scales. Therefore, it is advisable to apply appropriate normalization techniques before training these models. Another commonly used method for data rescaling is min–max scaling, which adjusts data to a fixed range, typically [0, 1]. This is achieved by subtracting the minimum value from all data points and then dividing it by the range, which is the difference between the minimum and maximum values.

For spectral descriptors like SOAP, however, the situation is different. Each column in the SOAP descriptor represents an element in a coherent spectral series, where the relative magnitudes encode critical structural or chemical information. Applying feature-wise scaling methods such as *z*-score normalization or min–max scaling can disrupt these relationships, leading to the loss of physical meaning. Therefore, no scaling should be applied by default (scaler = none) to preserve the integrity of SOAP descriptors. Alternatively, dimensionality reduction methods like Kernel PCA with an RBF kernel can be employed to reduce descriptor size while retaining the most relevant spectral features.

### 3.3 Dimensionality reduction

Dimensionality reduction is a data analysis technique that simplifies complex datasets by reducing the number of features or variables while retaining essential information. Chemical systems, like molecules, are inherently high-dimensional due to the multitude of properties, descriptors, and interactions involved. For instance, a molecule can be characterized by features such as bond lengths, angles, dihedrals, and even electronic properties. When handling such high-dimensional data, which may include hundreds or thousands of variables, visualizing and analyzing the information effectively becomes challenging. Dimensionality reduction is valuable in two key ways: it helps with data visualization by transforming high-dimensional data into a more manageable format,^53^ and it reduces redundancy by removing correlated or redundant features.^54^ Reducing redundant features can enhance the efficiency and performance of subsequent data use. Below, we summarize some of the most common dimensionality reduction methods applied to model chemical systems and available in ULaMDyn.

#### 3.3.1 PCA (principal component analysis)

PCA^55^ is a widely used linear multivariate statistical technique for dimensionality reduction, valued for its interpretability.^56^ As a matrix factorization method, PCA identifies patterns and extracts critical features from high-dimensional datasets by transforming the original variables into a new set of orthogonal, uncorrelated variables known as principal components. This process achieves a lower-dimensional representation of the data while retaining as much of the original information as possible. PCA is particularly effective when the primary variations in the data are linear, making it a good starting point for exploratory data analysis.

#### 3.3.2 t-SNE (t-distributed stochastic neighbor embedding)

t-SNE is a nonlinear dimensionality reduction technique used in various fields, including molecular systems.^57^ Unlike PCA, the dimensions obtained with t-SNE do not have a straightforward interpretation, so it is primarily used for visualization or exploratory data analysis. t-SNE maps high-dimensional data into a lower-dimensional space (embedding) based on similarities between data points, calculated using a Gaussian kernel or a Student's *t*-distribution. It constructs a neighborhood graph where each node is connected to its nearest neighbors, forming local relationships. The parameter perplexity controls the balance between preserving local and capturing global structures; higher perplexity values capture global relationships, while lower values emphasize local structure. t-SNE then creates probability distributions for both high- and low-dimensional spaces and uses an optimization algorithm, such as gradient descent, to iteratively adjust the positions of the mapped points until the divergence between the distributions is minimized or the maximum number of iterations is reached. The resulting embedding can be visualized in scatter plots or further analyzed for insights into the data structure.

#### 3.3.3 ISOMAP (isometric feature mapping)

ISOMAP is a nonlinear dimensionality reduction technique that extends classical multidimensional scaling (MDS) by incorporating geodesic distances.^58^ It is designed to uncover the underlying manifold structure of high-dimensional data. ISOMAP works by first constructing a neighborhood graph of data points using a method like k-nearest neighbors or ε-neighborhoods. It then calculates the shortest paths between all pairs of points in this graph, approximating the geodesic distances on the manifold. These distances are used to perform classical MDS, resulting in a lower-dimensional representation that preserves the intrinsic geometric structure of the data. ISOMAP is particularly useful when the data lies on a nonlinear manifold and is effective for capturing global structures in the data. It provides a way to visualize complex, high-dimensional data in a more interpretable form.

### 3.4 Clustering

Clustering methods are commonly used to make sense of these large datasets, helping to organize chemical systems into subgroups with shared electronic properties or spatial configurations. Unlike dimensionality reduction, which compresses data into a smaller set of critical components and may create artificial groupings, clustering identifies natural subgroups in the original data space. This preserves the intrinsic relationships and structures within the data, making it particularly useful for identifying families of molecules with similar electronic properties or revealing patterns in chemical composition and spatial configurations essential for understanding molecular interactions and reactivity. A well-tuned clustering algorithm distills large volumes of data into a manageable number of qualitatively distinct categories, facilitating data visualization and exploration. This approach also improves the efficiency of further computational and experimental studies by enabling a more focused analysis of nonadiabatic dynamics, thereby enhancing our understanding of excited-state processes and transitions.

In ULaMDyn, several algorithms are available for clustering analysis, each differing in its interpretation of what constitutes a cluster and how clusters are identified. The clustering algorithms provided include:^59–62^

• *K*-Means clustering: this algorithm partitions data into a predefined number of clusters by minimizing the sum of squared distances between data points and their respective cluster centroids.^63^ Because *K*-means assumes an isotropic data distribution per cluster (equal variance in all directions), this method tends to work better on datasets with inherent globular or spherical cluster shapes. Each data point is assigned to the nearest cluster in a rigid, non-overlapping manner, resulting in distinct Voronoi cells that define the cluster boundaries.

• Gaussian Mixture Model (GMM): this method can be seen as a generalization of *K*-means in the sense that each cluster is described by a Gaussian distribution, allowing for more flexible cluster shapes, such as ellipses, with varying size, orientation, and covariance.^64,65^ Unlike *K*-means, which uses hard assignments, GMM employs a soft (probabilistic) approach, where each data point is assigned a probability of belonging to multiple clusters. This results in a more flexible clustering approach, as data points can partially belong to different clusters. GMM is particularly suited for datasets where clusters overlap or exhibit complex shapes, making it a more versatile method for capturing non-spherical structures. Because GMM learns the underlying data distribution, it can also be used as a generative model to sample geometries from specific regions of the NAMD trajectory space.

• Hierarchical agglomerative clustering: this method utilizes a bottom-up approach that starts with each data point as a separate cluster and iteratively merges the closest pairs of clusters until a single cluster remains.^66^ This process creates a hierarchical structure called a dendrogram, which can be cut at different levels to obtain clusters of varying granularity. Therefore, this agglomerative clustering does not require a predetermined number of clusters. The method does not assume any specific cluster shape, making it well-suited for complex data distributions.

• Spectral clustering (equivalent to kernel *K*-means): this method extends *K*-means by first representing the data as a graph, where each data point is a node, and edges represent pairwise similarities between points.^67,68^ Spectral clustering then uses the eigenvalues of the graph's Laplacian matrix to transform the data into a lower-dimensional space, capturing the critical connectivity patterns in the data. This transformation allows for the identification of complex, nonlinear clusters that *K*-means might miss. Unlike *K*-means, which operates directly in the original input space and assumes spherical clusters, spectral clustering leverages the graph structure to uncover clusters of arbitrary shape. By relying on a similarity matrix rather than distance measures, spectral clustering is particularly well-suited for data where distance-based methods are less effective.

For dimensionality reduction and clustering, ULaMDyn uses existing implementations from scikit-learn.

## 4 Walkthrough example

In this section, a walkthrough example of installing and using ULaMDyn is provided. The entire clustering or dimensionality reduction analysis workflow is automated by ULaMDyn, starting with data collection and the conversion of molecular geometries into descriptors. The selected unsupervised learning algorithms are then applied to the descriptor space. Additionally, various postprocessing statistical analyses are conducted by grouping the data according to the cluster labels generated by the clustering process. All these steps can be executed *via* a command-line interface (CLI) or by customized Python scripts by importing the ULaMDyn modules. In the following subsections, this pipeline will be broken down, demonstrating how each step can be carried out within a Python framework using ULaMDyn. A Python notebook of this example can be found at https://gitlab.com/light-and-molecules/ulamdyn_paper-2025.

### 4.1 Installation

**Fig. 2:**
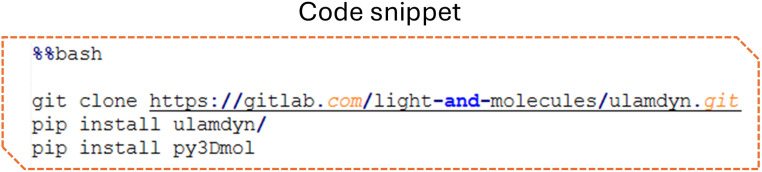
Installation code snippet.

ULaMDyn can be conveniently installed from its repository, along with all necessary dependencies (Fig. 2). Additionally, several packages are required for visualization purposes, ensuring comprehensive data analysis and representation.

### 4.2 NAMD dataset

In this example, fulvene serves as a photoactive molecule undergoing structural transformation during nonadiabatic dynamics simulations initiated from the first excited state.^69^ Fulvene is adopted here because it is a typical test molecule used for the development of methods and benchmarks in NAMD.^70–72^ The reason for its popularity stems from its small size (allowing quick simulations even at fully correlated levels) and ultrafast dynamics (all relevant events are completed in less than 100 fs).^71^ Fulvene has also been shown to be the multidimensional analogous of the popular Tully III analytical 1D model.^73^

Fulvene dynamics is characterized by ultrafast nonadiabatic processes involving S_1_ ↔ S_0_ recurrences at an extended conical intersection seam.^74^ The molecule exhibits fast decay to the ground state (S_0_) followed by periodic recurrences to the first excited state (S_1_). In fulvene dynamics, the most relevant coordinates include the torsional angle around the C–CH_2_ bond and the bond length between these carbon atoms. These coordinates are critical because they define the molecule's structural evolution as it moves toward the conical intersection at different regions of the crossing seam.

The Newton-X CS (classical series) program was employed to propagate 200 surface hopping trajectories up to 60 femtoseconds with a time step of 0.1 fs. The CAS(6,6)/6-31G* method was utilized to calculate the quantum chemical properties for the two electronic states (S_0_ and S_1_).^70^ The complete dataset can be downloaded from figshare.com/articles/dataset/Fulvene_DC-FSSH/14446998?file=27635412. For the sake of time, only a subset of the trajectories (50 trajectories) was selected for this example.

After downloading and unpacking the NAMD data, navigate to the working directory with the 50 TRAJ folders, each containing the simulation results. ULaMDyn will automatically detect the available trajectories and extract all the necessary information for further analysis.

### 4.3 Data import and inspection

The first step in the pipeline for analyzing nonadiabatic molecular dynamics (MD) data involves gathering relevant quantities, such as molecular geometries, potential energies for each electronic state, kinetic energy, energy gradients, and oscillator strengths, computed for each trajectory. In Newton-X, this information is typically provided in text files located within each TRAJ#/RESULTS folder. Built-in classes are included in ULaMDyn and are specifically designed to aggregate these data, storing them in Python objects for straightforward manipulation. The usage of these data collection classes within a Python environment is demonstrated in the following section.

ULaMDyn reads the output of the dynamic only once and converts it into Pandas data frames, saving it in CSV format for future analysis. Leveraging the intrinsic parallelization of Pandas and Numpy, it efficiently processes datasets. Tests with typical AIMD simulations (100 trajectories, 10k steps, 12 atoms) indicated memory usage of up to 3 GB and 1 hour for statistical analyses of all properties. Larger systems (*e.g.*, Rhodamine 110 with 39 atoms) doubled these requirements. While current analyses are manageable, ongoing efforts aim to further optimize performance and address challenges arising with larger datasets driven by advancements in machine learning potentials and semi-empirical methods.

#### 4.3.1 Running ULaMDyn as a command-line interface

Before we get started with the capabilities of ULaMDyn through Python API, it is important to highlight that ULaMDyn also provides a set of predefined functions accessible through a command-line interface. This simplifies workflows and enhances the overall user experience. Once ULaMDyn is installed, users can execute the wrapper script, 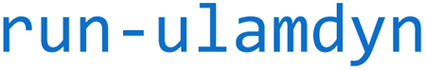, directly from the Linux terminal. Additional options can be specified with the command-line parser, which can be viewed by invoking the -help flag.

The wrapper script is executed from the main TRAJECTORIES (nx_traj_fulv) directory, which must include a reference geom.xyz file and a Newton-X input file inside the TRAJ1 subdirectory. By executing the command-line program, ULaMDyn will first generate structured datasets (such as flattened *XYZ* coordinates, *Z*-matrices, and quantum mechanical (QM) properties) in a CSV file containing all information collected from the output files of different trajectories. After that, the program can compute the basic descriptive statistics (mean, median, and standard deviation) for each dataset and export these results as separate CSV files. If additional options are specified *via* the command line, they will be incorporated into the workflow and executed accordingly.

#### 4.3.2 Geometries

**Fig. 3:**
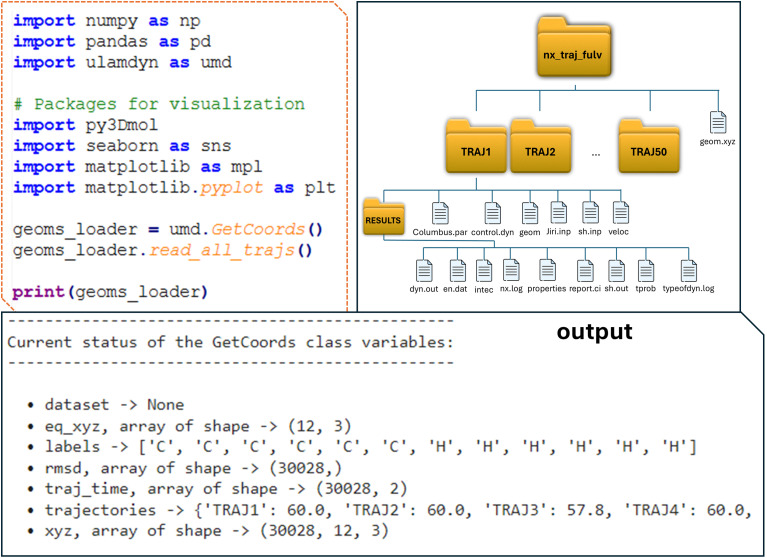
Setup for getting the dataset and extracting geometries from Newton-X trajectories using ULaMDyn. (Left) Directory structure with the nx_traj_fulv folder containing individual TRAJ# subdirectories. (Right) Code snippets show ULaMDyn's GetCoords class used to load molecular geometries, displaying the status of class variables.

The data collection process in ULaMDyn is organized in a modular fashion, with a specific class called 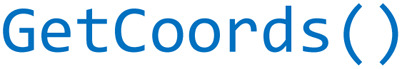 dedicated to reading geometries from Newton-X trajectories. This class simplifies the process by automatically extracting all the molecular geometries from the available trajectories. When executed, it systematically reads all the geometries provided in the standard Newton-X output (dyn.out or .h5), ensuring comprehensive data acquisition. In the subsequent printout (Fig. 3), the 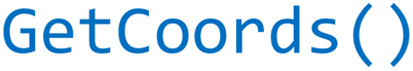 object lists all available trajectories detected in the folder, along with the corresponding atomic labels, which can be used for the generation of SOAP descriptors. These labels can be utilized, for example, to calculate mass-weighted coordinates when necessary. Additionally, the total number of geometries loaded from all these trajectories is displayed (Fig. 3 bottom).

**Fig. 4:**
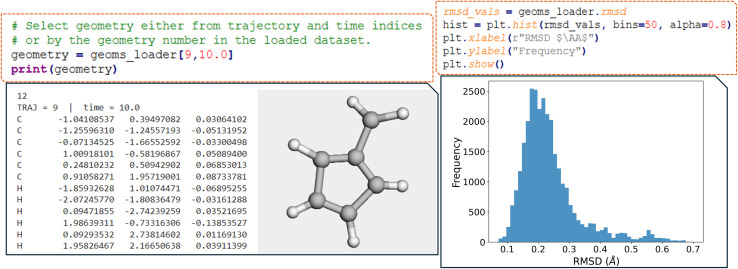
(Left panel) Selection of a molecular geometry at a given time using ULaMDyn. The script snippet demonstrates how to access the *XYZ* coordinates of a fulvene molecule from trajectory 9 at the 10.0 fs time step. The output shows the atomic coordinates (in Å) and a 3D visualization of the molecule's structure. (Right panel) Histogram of the RMSD of geometries with respect to the equilibrium geometry.

After running the read_all_trajs function (see Fig. 3), the processed geometries are conveniently stored as an object attribute, along with their corresponding trajectory and time step indices, allowing for easy selection of a specific geometry from a given trajectory at any time step (geoms_loader). This built-in indexing of the geoms_loader object streamlines geometry selection for rapid inspection and analysis of the structures within the simulation data. For example, the *XYZ* of trajectory 9 at 10.0 fs can easily accessed for molecular visualization by passing these two indices as arguments (Fig. 4).

By providing a reference geometry file, geom.xyz, in the working directory, the software automatically computes the root mean square deviation (RMSD) between each frame of the trajectories and this reference geometry, typically the ground state minimum. In the example above, the resulting RMSD distribution reveals that most geometries are clustered around an RMSD value of approximately 0.21 Å, indicating significant deviations from the ground state. A few geometries exhibit even more significant distortions, as shown by the long tail in the distribution (Fig. 4).

#### 4.3.3 Energies and other properties

In ULaMDyn, several utility functions have been designed to enable the consistent extraction of various properties from Newton-X simulation output files. For instance, energy information can be retrieved by reading the relevant files and compiling a dataset. Quantum mechanical quantities, such as potential energies and oscillator strengths, are collected from the Newton-X outputs using the 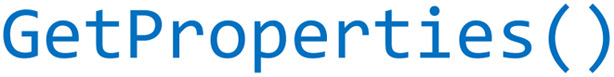 class. The properties dataset includes columns for trajectory identifiers and time steps, which are essential for uniquely identifying each data point. Consistent identifiers across different datasets, like those for molecular geometries or RMSD values, are crucial for accurate data merging and analysis.

Typically, this dataset includes columns for total energy (Total_Energy), hopping event from excited to ground state (Hops_S21), ground state energy (S1), and energy gaps (DE21) and back hopping event (Hops_S12) between states when multiple states are considered. It also includes an indicator for hopping geometries, where a value of ‘0’ denotes a no-hopping and ‘1’ indicates a hopping event. This setup facilitates easy filtering of hopping geometries for further analysis. Additionally, users can extend the dataset by incorporating other calculated quantities, such as state populations, which are typical outputs in nonadiabatic molecular dynamics simulations.

**Fig. 5:**
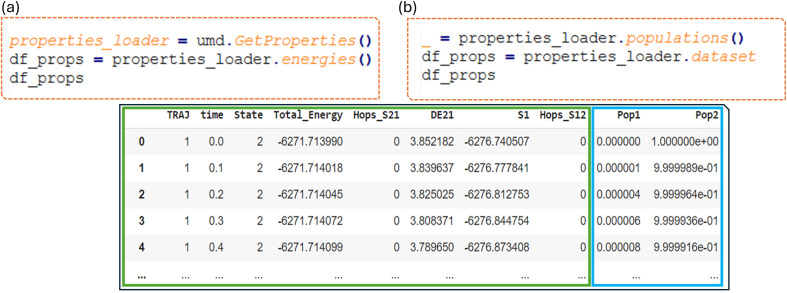
Example of using ULaMDyn's GetProperties class: (a) code snippet retrieves potential energies and dataset details in the green rectangle with columns representing the current state (State), total energy (Total_Energy), hopping event from S2 to S1 (Hops_S21), energy gap (DE21), ground state energy (S1), and back hopping event (Hops_S12); (b) additional code snippet incorporates state population data with columns representing ground state population (Pop1) and excited state population (Pop2), shown in the blue rectangle, for analyzing nonadiabatic transitions in fulvene.

In the case of fulvene dynamics (Fig. 5), the potential energy of the ground and first excited states, the oscillator strength corresponding to the transition between these states, and the MCSCF coefficients of the CAS wave function are available and shown in the columns. Additionally, a function is provided to collect the % of the state's population as computed by Newton-X (Pop1 and Pop2).

### 4.4 Generation of geometric descriptors

The 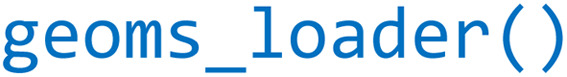 object in Python encapsulates the attributes of the collected geometries from Newton-X outputs. The ‘xyz’ attribute of this object can be accessed to retrieve all stored geometries. The data is structured as an array, with dimensions corresponding to the number of samples (representing the total number of geometries in the simulation), the number of atoms, and the three spatial coordinates (*x*, *y*, and *z*), thus providing a comprehensive dataset for further analysis.

**Fig. 6:**
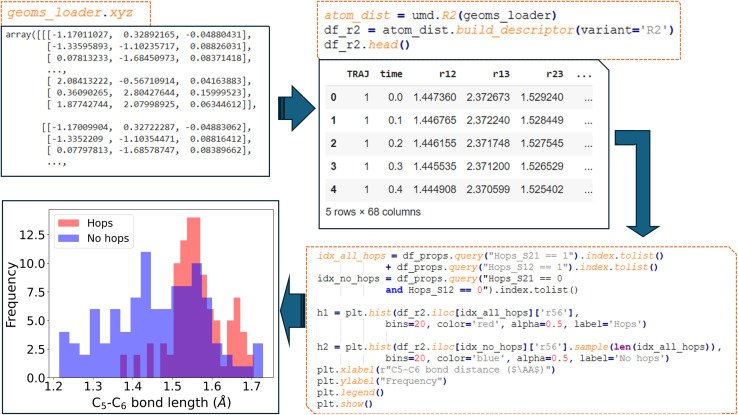
Workflow demonstrating the use of ULaMDyn to generate descriptors and visualize results. The left panel shows the raw coordinates loaded from NAMD simulations. These coordinates are used to build an R2 descriptor (df_r2), as seen in the top-right panel. The bottom-right panel presents the code to create a histogram comparing C_5_–C_6_ bond distances for geometries with and without hopping events, with the resulting histogram displayed in the bottom-left panel.

As mentioned earlier, ULaMDyn provides three classes of symmetry-aware descriptors (translational and rotational invariant) based on molecular geometries: the pairwise atom–atom distances (R2 family of descriptors) and the Z-matrix representation. These descriptors are computed by processing the NAMD molecular geometries stored in the 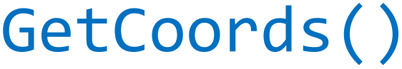 object. In the example provided below (Fig. 6), the function 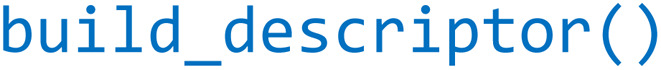 within the R2 descriptor class is used to return a Pandas data frame object with the descriptor calculated for all geometries of each NAMD trajectory. Additional flexibility for representing molecular structures in a manner invariant to translational and rotational transformations is offered by other variants of the R2 descriptor supported by this function.

An important application of this properties dataset is to distinguish between hopping and non-hopping geometries. By filtering the dataset accordingly, the distribution of specific bond distances (such as the C_5_–C_6_ bond in fulvene) can be plotted for hopping *versus* non-hopping geometries. For fulvene, the analysis revealed that the distribution of hopping geometries (represented by red bars) has shifted towards larger C_5_–C_6_ bond distances (Fig. 6). This observation is aligned with the understanding that, at the beginning of the dynamics, the bond stretching in fulvene often leads the system towards a conical intersection, facilitating nonadiabatic transitions.^69^ The structural differences between hopping and non-hopping geometries are underscored by the distinct distributions, with hopping geometries predominantly concentrated around a bond distance of approximately 1.55 Å.

**Fig. 7:**
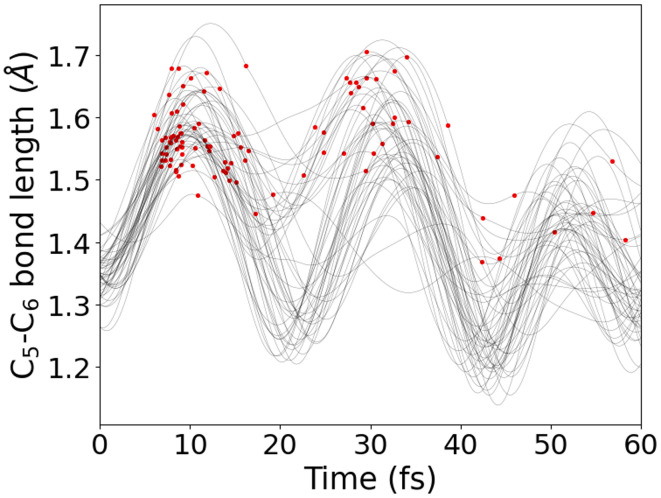
Stretching of C_5_–C_6_ bond length of fulvene for 50 trajectories. Points in red indicate the hopping events, which mainly occur at the maximum bond length.

An alternative method to inspect the dataset and gain insights into its behavior is by plotting bond distances (*y*-axis) as a function of time (*x*-axis) (Fig. 7). This type of analysis, part of the standard routine for understanding nonadiabatic molecular dynamics (NAMD) simulations, helps visualize the evolution of geometrical features such as bond stretching and contraction. Additional information, such as hopping geometries, can be overlaid on this plot. Notably, in this analysis, most hopping points, marked by red indicators, cluster around the maxima of the oscillations in the bond distance. ULaMDyn serves as a tool to facilitate this type of interaction with NAMD simulation results, allowing users to extract and interpret key information efficiently. While these analyses are routine, they lay the groundwork for more advanced unsupervised learning methods, which automate the extraction of deeper insights from high-dimensional data.

Up to this point, the focus has been on exploring the dataset to identify key features. This exploratory phase allows for experimentation and various tests to understand the dataset better. Depending on the specific requirements of the analysis, this level of exploration may already provide sufficient insights.

### 4.5 Dimensionality reduction

In the R2 dataset of fulvene, which includes all non-equivalent atom–atom distances, it is noted that 68 columns are initially present, two of them corresponding to the index tuple of trajectory number and time. Therefore, the descriptor has 66 variables, with the molecular geometries represented in a high-dimensional vector format. Direct visualization of this correlated high-dimensional data is not feasible; however, dimensionality reduction techniques can be applied to reduce the 66 columns to two, allowing the representation of the geometry evolution in a simple 2D scatter plot.

**Fig. 8:**
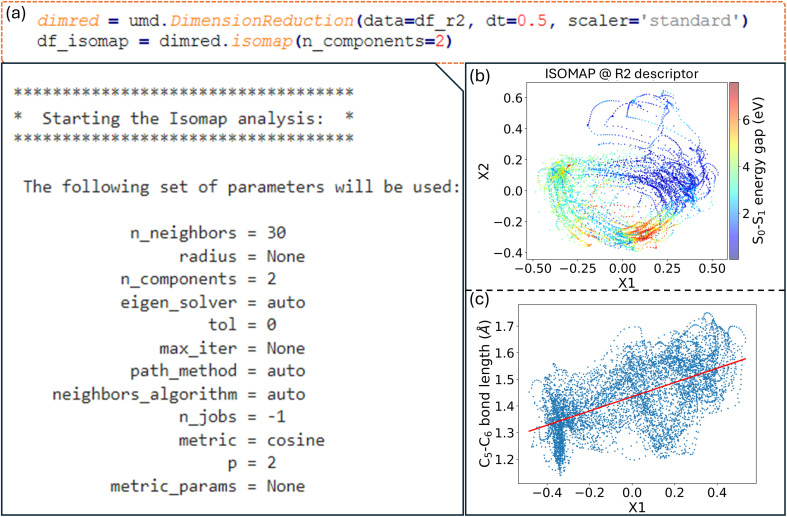
(a) Dimensionality reduction using ISOMAP in ULaMDyn, with the parameters for the ISOMAP analysis; (b) the 2D ISOMAP embedding of the R2 descriptor, with the S_0_–S_1_ energy gap color-coded; (c) the relationship between the first ISOMAP component and the C_5_–C_6_ bond distance.

In ULaMDyn, the 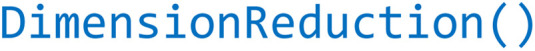 class is used to perform dimensionality reduction on the descriptor dataset provided as input. To minimize the correlation between consecutive geometries, the dataset can be resampled at larger time intervals. For instance, a time step of 0.5 fs, five times larger than the time step used to generate the NAMD trajectories, is employed to reduce temporal correlation, enhancing the algorithm's ability to identify meaningful patterns. The 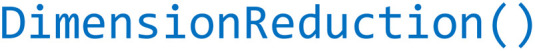 class provides access to several methods for dimensionality reduction with ISOMAP, a nonlinear technique, being utilized here for demonstration. This method has proven particularly effective in identifying critical active coordinates that dominate photophysical processes in nonadiabatic simulations of complex molecular systems.^32^ The dataset is reduced from 66 dimensions to two by the ISOMAP method for visualization. A neighborhood graph with a specified number of neighbors (*e.g.*, 30 Fig. 8a) is constructed by the ISOMAP method, and this graph is used to map high-dimensional data into a lower-dimensional space.

A distribution is revealed by the plot shown in Fig. 8b, where points with similar properties (*e.g.*, energy gaps) are clustered together despite this information not being explicitly provided as input to the method. Important geometries and regions of interest within the dataset are easily identified through this visualization, which condenses information on independent NAMD trajectories. Although the selection of molecules based solely on properties such as the energy gap is possible, it is noted that this approach may not always capture nuanced differences in geometry. A more comprehensive view is provided by the dimensionality reduction diagram (Fig. 8b), which highlights distinct regions and aids in the selection of significant molecular geometries for further analysis.

In nonlinear dimensionality reduction, the relationship between the embedded dimensions and the original features is typically complicated in determining their contribution to clustering patterns. An alternative to gaining intuition about these relationships is to plot each geometrical feature of the molecules against the embedded dimensions. For example, plotting the first reduced dimension, X1, against the C_5_–C_6_ bond distance reveals a positive correlation (Fig. 8c). In the case of fulvene, this indicates that the X1 coordinate effectively captures the bond stretching trend involving the CH_2_ group, which is expected to be one of the key motions for driving the system throughout the conical intersection. This interpretation strategy can be applied to many other quantities to further explore the relationships between geometrical features and the reduced dimensions.

**Fig. 9:**
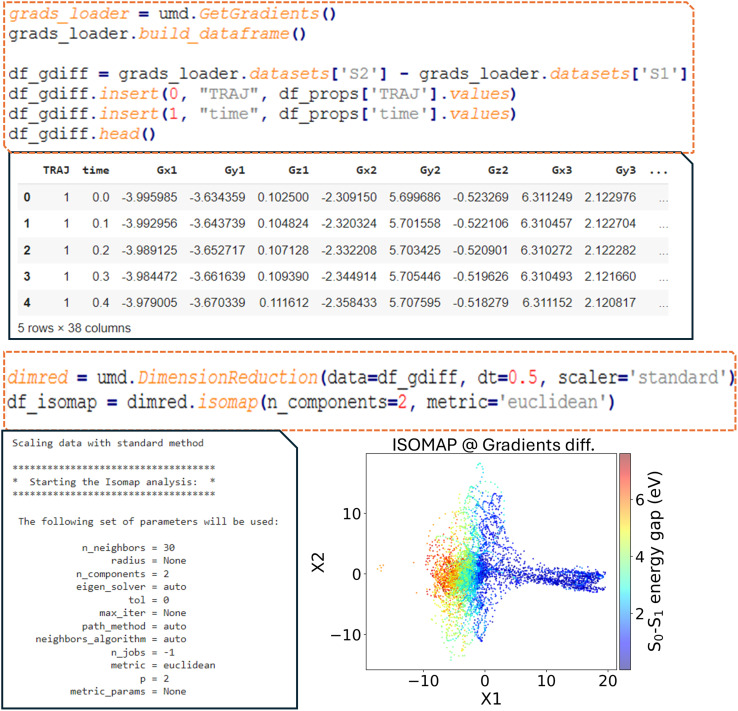
Dimensionality reduction performed on the difference between gradients, with a 2D ISOMAP embedding of the gradient differences shown. Gx1, Gy1, and Gz1 represent the differences in gradients along the *x*, *y*, and *z* directions for the first atom, respectively, between the first excited singlet (S_1_) and the ground state (S_0_) and so on for the subsequent atoms. The S_0_–S_1_ energy gap is color-coded.

Since the analysis performed here is a postprocessing step on the NAMD simulation data, the analysis is not limited to examining the molecular geometries. Any quantum chemical information available from the simulations can be used as a descriptor for unsupervised learning analysis. For instance, crucial information on how rapidly geometries can evolve during dynamics is provided by the energy gradient matrices of each potential energy surface. Fig. 9 shows the difference between the energy-gradient matrices of the S_1_ and S_0_ states as an example. Although the ISOMAP diagram derived from the gradient difference descriptors looks different from the one obtained with the R2 descriptor (geometry-based), one can observe that geometries with small and large energy gaps between the S_0_ and S_1_ states still appear as clearly distinct groups in the plot.

### 4.6 Clustering

Up to this point, the focus has been on exploring the dataset to understand its main features. Now, clustering analysis will be applied to the features without dimensional reduction.

A common question is whether dimensional reduction should be performed before clustering. This decision depends on the specific context. When dealing with large or sparse vectors representing each molecule, dimensionality reduction can be advantageous. Reducing dimensions often helps eliminate unnecessary or correlated components, making it easier to compare data points; it is often used for visualization purposes. On the other hand, the compact representation resulting from dimensionality reduction can introduce artificial clusters, as distances between points may be shrunken compared to the original space, potentially distorting the true relationships in the data. In the case of fulvene, the molecule is relatively small, and the vectors used for representation are not very large. Therefore, clustering will be applied directly to the descriptors without any prior dimensional reduction. Dimensional reduction can still be used afterward to visualize how the clustering algorithm identified different groups within the dataset.

**Fig. 10:**
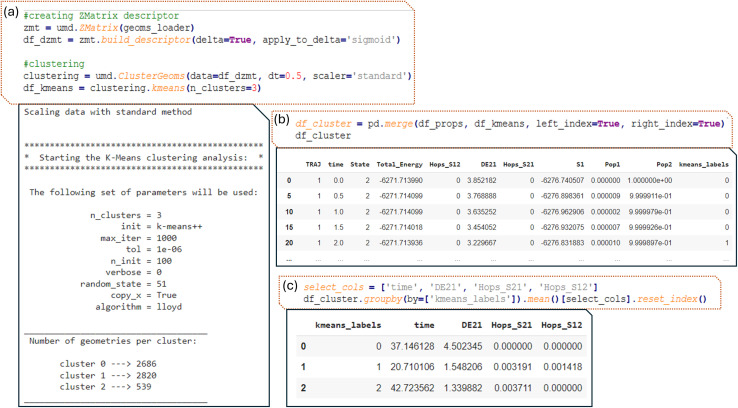
(a) The Z-matrix descriptor is created for geometries, followed by clustering using *K*-means (with 3 clusters). The parameters for *K*-means are detailed, showing the number of geometries in each cluster; (b) the clustered data is then merged with properties data (df_props); (c) the average values of selected properties (DE21, Hops_S21, Hops_S12) are computed for each cluster.

The process begins with the creation of our descriptor, the delta Z-matrix, in this case. This involves subtracting the Z-matrix of the ground state from the geometry of each frame of the dynamics. Additionally, a nonlinear function is applied to focus on points that are not outliers, ensuring that the algorithm identifies those that are more like the ground state. The geoms_loader object, which contains geometries from different trajectories, is passed as input. The geometries are read by ULaMDyn, and the descriptor is created accordingly (Fig. 10a).

Next, geometries are grouped by similarity in the descriptor space using the clustering module of ULaMDyn (Fig. 10a), which contains different methods similar to the dimensional reduction module. The data set, represented by the descriptor, is provided as input. To reduce the size of the data set and eliminate some correlations, a specific time slice can be given, or a fixed number of samples selected randomly (such as 1000 geometries from the original data set) can be specified.

Based on the color pattern of the ISOMAP plot in Fig. 9 (red for large, green for intermediate, and blue for small energy gaps), a reasonable guess for the number of clusters in this demonstration would be three. In cases where this information is not readily available, ULaMDyn offers a built-in function that combines the Silhouette score^75^ and Caliński–Harabasz^76^ metric to determine the optimal number of clusters. This feature can be accessed by setting the number of clusters parameter to ‘best’.

Once the clustering object is created, the *K*-means algorithm is applied using the command ‘
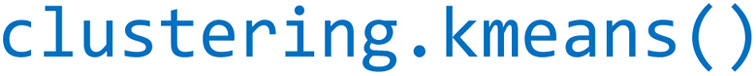
’. Because the data set is stored within the clustering object, multiple algorithms can be run and easily compared using the same input data set. In this example, the *K*-means algorithm is used for clustering. To switch to a different algorithm, one needs to change ‘kmeans’ to another algorithm, such as ‘hierarchical’ for hierarchical clustering.

In this example, the *K*-means algorithm split the data into three clusters labeled 0, 1, and 2, where cluster two contains significantly fewer geometries compared to the others. Upon closer inspection and analysis, we observe that this smaller cluster contains geometries related to CH_2_ rotations. This is consistent with the finding that such rotated geometries are less frequently visited during the dynamics compared to other points. Consequently, the smaller cluster size reflects the lower occurrence of these rotated geometries.

The data set (df_cluster) containing the *K*-means clustering labels is straightforward. Each label corresponds to a specific row in the data set, indicating which cluster the geometry belongs to. By concatenating the *K*-means labels with the properties dataset, it becomes clear which geometries belong to each cluster, revealing, for instance, that geometry with a large energy gap (around 3.8 eV) belongs to cluster zero, among other insights (first line of output in Fig. 10b). Python simplifies the manipulation of this data, allowing for efficient grouping and analysis of the points within each cluster. Thus, by grouping all rows corresponding to a given cluster, statistics such as the mean value of a given property can be calculated (Fig. 10c). For example, cluster one has an average energy gap of 1.55 eV and contains many hopping geometries. In contrast, cluster zero has essentially no hopping points. This type of analysis enables a deeper understanding of the characteristics within each cluster.

**Fig. 11:**
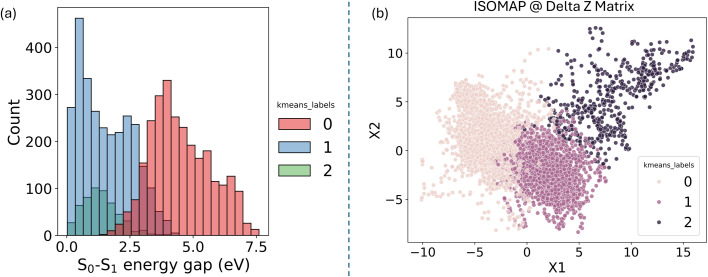
(a) Histogram displaying the distribution of the S_0_–S_1_ energy gap (in eV) across different clusters identified by *K*-means clustering. Each color represents a different cluster label. (b) ISOMAP projection of the delta Z-matrix data, where the points are colored according to their respective *K*-means cluster labels, showing the separation of clusters in the reduced dimensional space.

The distribution of the energy gap for each cluster can be visualized using a histogram (Fig. 11a). This helps illustrate the differences in the energy gap across the clusters. Despite the energy gap not being included in the descriptor used for clustering, its distinct distribution among clusters underscores the effectiveness of the data partitioning algorithm, which serves as a validation step, confirming that the clustering results are meaningful. For example, clusters zero and one exhibit distinct energy gap distributions, highlighting their unique characteristics. On the other hand, clusters one and two show some overlap in the region of small energy gaps. This observation aligns with the findings, which identified two different types of geometries associated with hopping events: one related to bond stretching and another linked to the rotation of the CH_2_ groups.

To further assess the effectiveness of the clustering algorithm, dimensionality reduction was applied to the differential Z-matrix data set. The two primary components were plotted, and the points were colored according to their cluster assignments to visually evaluate the clustering results (Fig. 11b).

In *K*-means clustering, the partitioning is known as a “hard partition,” meaning each point is strictly assigned to a single cluster. This method does not allow for probability distributions across clusters as in the Gaussian Mixture model. As a result, *K*-means may struggle with data sets where clusters overlap significantly, as a straight boundary tends to be drawn between them. This limitation can lead to less defined clusters, especially when the clusters are not well-separated in space. This behavior is evident in the plot shown in Fig. 11b, where cluster two is distinctly different, but clusters zero and one could potentially be merged into a single cluster. This ambiguity suggests that using a different clustering algorithm, such as hierarchical clustering, might yield different results, potentially combining these clusters into one. However, with *K*-means, the data set will consistently be partitioned into distinct parts, irrespective of such nuances.

With the cluster labels now established, the next step involves analyzing what these labels reveal in terms of the underlying chemistry. To do this, standard Z-matrices without any difference from the ground state are calculated to obtain bond lengths, angles, and dihedrals. The objective here is to investigate whether the clustering correctly distinguishes the data according to the two degrees of freedom that are expected to be most significant. In this particular case, the importance of these degrees of freedom is already known, simplifying the analysis. However, in scenarios where the chemistry of the system is less understood, it would be advisable to spend more time exploring the data beforehand. This exploration would help develop an intuition for how the variables are distributed.

**Fig. 12:**
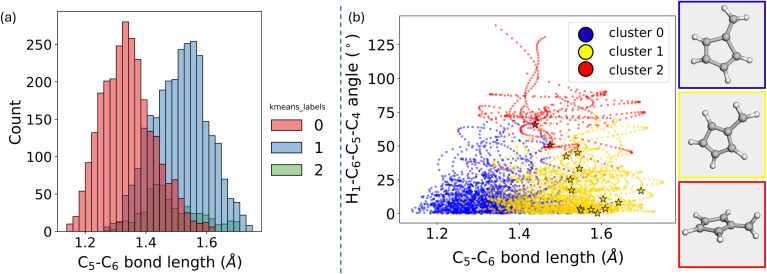
(a) Histogram showing the distribution of C_5_–C_6_ bond distances (in Å) categorized by *K*-means cluster labels. Each color represents a different cluster. (b) Scatter plot of the H_1_–C_6_–C_5_–C_4_ dihedral angle *versus* the C_5_–C_6_ bond length, with points colored according to their corresponding cluster. Representative geometries for each cluster are displayed alongside the plot.

Upon calculating the bond lengths and dihedrals, specifically the C_5_–C_6_ bond distance, significant differences are observed between clusters 0 and 1 (Fig. 12). The mean values of these distributions are noticeably different. Cluster 2 shows more dispersed values, particularly in the green distribution, which likely corresponds to the 90° rotation discussed earlier. The blue cluster primarily consists of geometries with shorter bond lengths. In contrast, the yellow cluster includes geometries with an extended C_5_–C_6_ bond length. The markers in this plot correspond to the hopping geometries. It becomes evident from this analysis that some hopping geometries belong to cluster 1 (yellow). In contrast, a few others, represented by red star markers, belong to cluster 2.

By analyzing the hopping geometries within cluster 2, it has been confirmed that they correspond to the 90-degree rotated structures. These kinds of structures are responsible for less than 5% of the decay population of fulvene.^69^ Meanwhile, the hopping geometries in cluster 1 are associated with an elongated C_5_–C_6_ bond length. Additionally, the ring analysis module of ULaMDyn can be used to assess the puckering of rings.^77^

## 5 Conclusions and outlook

In this work, we introduced ULaMDyn, a robust and flexible Python package for analyzing nonadiabatic molecular dynamics (NAMD) simulations using unsupervised learning techniques. ULaMDyn integrates seamlessly with Newton-X and offers a complete pipeline for processing, reducing, and clustering high-dimensional molecular datasets, enabling researchers to uncover hidden patterns and critical molecular transitions. Through the case study of fulvene dynamics, we demonstrated its ability to identify critical geometries and provide insights into nonadiabatic transitions.

The automated nature of ULaMDyn streamlines the traditionally manual and labor-intensive task of postprocessing NAMD data, making it highly scalable for large systems and long timescale simulations. Beyond its current capabilities, the package is set to incorporate more advanced descriptors like MBTR and clustering techniques like DBSCAN, further enhancing its applicability to diverse molecular systems. With these upcoming features, ULaMDyn has the potential to significantly broaden its impact, facilitating the study of complex excited-state processes in areas ranging from photochemistry to materials science.

ULaMDyn has been designed to be the principal analysis tool for the many programs composing the Newton-X platform. However, users of other NAMD programs can also profit from the ULaMDyn capabilities by simply rewriting their results in the native Newton-X format, which is a matter of trivial scripting and postprocessing.

## Data availability

The code used for this work is available at https://gitlab.com/light-and-molecules/ulamdyn.git (tag 1.1.1). This program version, data, and the notebook used for the walkthrough example can be downloaded from Zenodo at https://doi.org/10.5281/zenodo.14624416. The data and the notebook used for the walkthrough example are also available at https://gitlab.com/light-and-molecules/ulamdyn_paper-2025. More information about ULaMDyn, including full documentation, can be found at https://ulamdyn.com. A video tutorial of ULaMDyn is also available on YouTube at https://www.youtube.com/watch?v=yMDUKhzipj0.

## Author contributions

Conceptualization: MPJ, MB; data curation: MPJ, JMT, SM; formal analysis: SM; funding acquisition: MB; methodology: MPJ; project administration: MB; software: MPJ, BCG; supervision: MB; validation: MTC, JMT, RSM, SM; visualization: BCG; writing – original draft: MPJ, MOB, BCG, MB; writing – review & editing: JMT, SM, MTC, MPJ, MOB, BCG, MB.

## Conflicts of interest

There are no conflicts to declare.
